# Cardiac Biventricular Metastasis From Renal Cell Carcinoma

**DOI:** 10.7759/cureus.10870

**Published:** 2020-10-09

**Authors:** Ahmad Nawid Latifi, Uzochukwu Ibe, Amit Arbune, Harriet Kluger, Lauren A Baldassarre

**Affiliations:** 1 Internal Medicine, Saint Mary's Hospital, Waterbury, USA; 2 Cardiology, Danbury Hospital, Danbury, USA; 3 Cardiology, Yale School of Medicine, New Haven, USA; 4 Hematology and Oncology, Yale New Haven Hospital, New Haven, USA; 5 Cardiology, Yale New Haven Hospital, New Haven, USA

**Keywords:** cardiac metastasis, cardiac tumor, renal cell carcinoma, metastatic renal cell carcinoma

## Abstract

Secondary cardiac tumors are much more common than primary tumors. Cardiac metastases from renal cell carcinoma (RCC) are rare and can present many years after the patient has been disease-free. We report the case of a 64-year-old man who had been treated for recurrent metastatic RCC. He presented with shortness of breath, and TEE (transthoracic echocardiography) revealed new biventricular hypertrophy and small-to-moderate circumferential pericardial effusion. Cardiac magnetic resonance demonstrated multiple lesions in both the ventricular walls, highly suspicious for metastasis. A tissue biopsy was obtained, which was inconclusive due to the small sample size. The patient's disease progressively worsened, and, subsequently, he died from cardiac and respiratory failure secondary to the underlying advanced metastatic disease. Cardiac metastasis from RCC is rare and has a wide range of presentations. Metastatic RCC tends to be resistant to chemotherapy and radiotherapy. Systemic therapy (immunotherapy, molecularly targeted agents) and surgery may have a role in these patients depending on the extent of disease and sites of involvement.

## Introduction

Metastatic cardiac tumors are over 20 times more common than primary tumors [1]. Renal cell carcinoma (RCC) is one of the tumors that rarely metastasize to the heart. Transthoracic echocardiography (TTE) is a good initial diagnostic imaging modality in a patient with suspected cardiac metastasis, along with electrocardiogram (EKG) and serum biomarkers. Cardiac magnetic resonance (CMR) is an ideal imaging modality for the diagnosis of cardiac metastasis, providing tissue characterization along with anatomical and functional information, helping clinch the diagnosis [2].

## Case presentation

**Figure 1:**
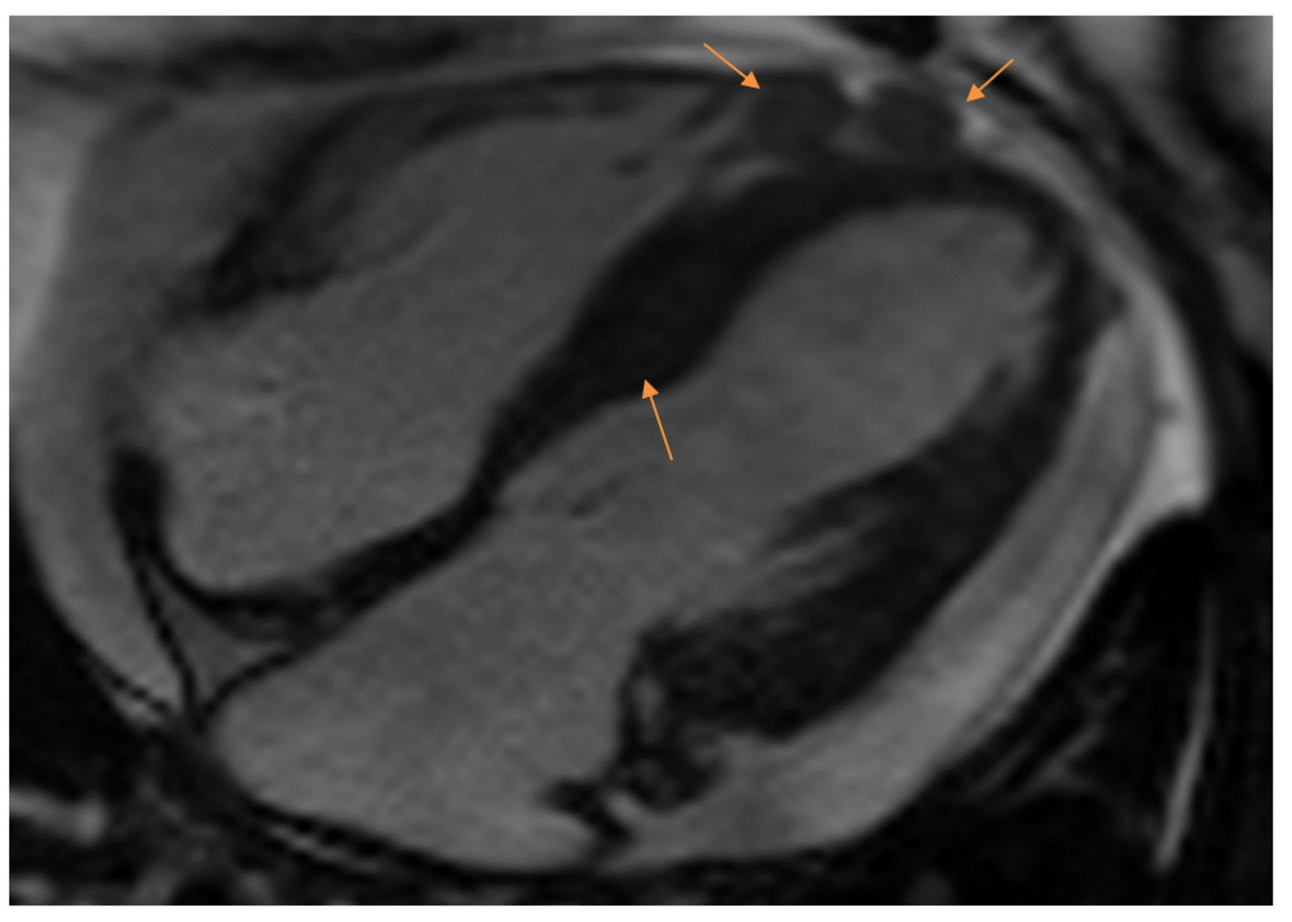
Four-chamber view of the heart as seen on cardiac MRI. Note the metastatic lesions in the right ventricular apex and the inferior interventricular septum, as indicated by the yellow arrows.

**Figure 2:**
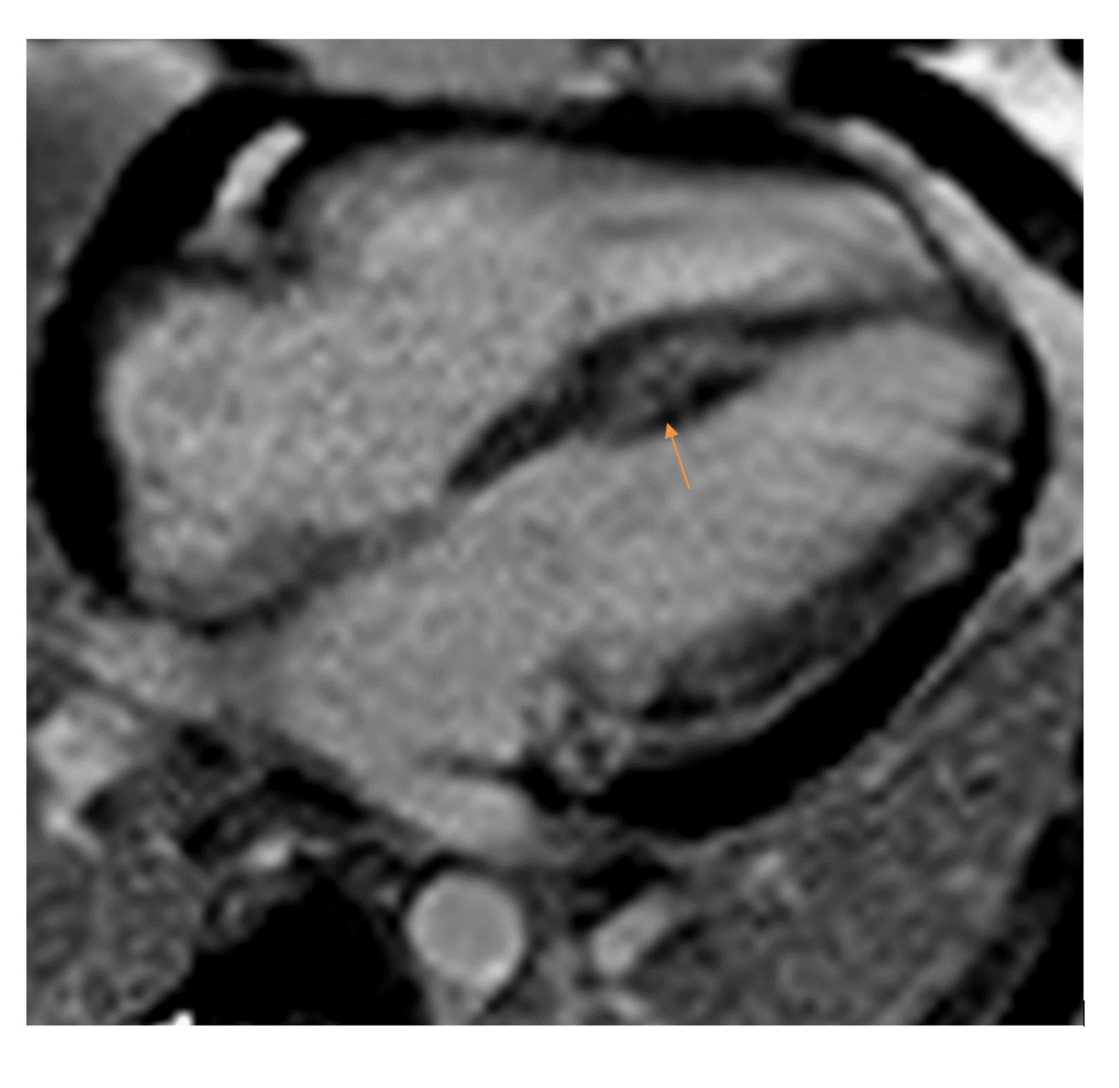
Delayed gadolinium enhancement imaging highlighting the presence of a metastatic lesion invading the inferior interventricular septum.

A 64-year-old man presented to the hospital in August 2018 with a chief complaint of worsening shortness of breath over the course of a week. He had coronary artery disease for which he underwent placement of with a drug-eluting stent to the left circumflex and obtuse marginal arteries about two years prior to presentation. He was diagnosed with clear-cell RCC in 2012 and underwent right nephrectomy. In 2014, on surveillance imaging, he was found to have lesions in the lung and mediastinal lymph nodes. Biopsy confirmed metastatic clear-cell RCC. He was enrolled in a clinical trial with atezolizumab plus bevacizumab, with a partial response. After two years, bevacizumab was discontinued due to worsening coronary artery disease, and after 14 months of atezolizumab alone, he was taken off the trial due to disease progression. In October 2017, he was enrolled on a trial of an anti-LAG3 antibody in combination with nivolumab, but did not respond. In January 2018, he started treatment with ipilimumab plus nivolumab on a clinical trial. A baseline TTE prior to starting ipilimumab plus nivolumab was unremarkable. In July 2018 (six months after starting nivolumab and ipilimumab), restaging CT scans of the chest, abdomen, and pelvis demonstrated further progression of his metastatic disease and mild pericardial effusion. A TTE revealed new severe concentric left ventricular (LV) hypertrophy, LV grade II diastolic dysfunction, dilated right ventricle with hypertrophy, pulmonary hypertension (PASP [pulmonary arterial systolic pressure] of 79 mm Hg), and a moderate circumferential pericardial effusion without tamponade physiology. CMR was obtained, which demonstrated masses in the basal inferior septum, mid inferolateral segment, and apical lateral segments (Figure 1). On delayed enhancement imaging, there was significant uptake of gadolinium-based contrast, suggestive of high vascular density, characteristic of RCC (Figure 2). To confirm the diagnosis, he underwent right heart catheterization with an endomyocardial biopsy. Pericardiocentesis was unsuccessful because of the limited effusion. The pathology specimen did not reveal malignant cells; however, metastatic disease could not be completely ruled out due to the small size of the biopsied specimen. Congo red staining for amyloid was negative. He was started on anticoagulation for the LV thrombus. His anti-neoplastic therapy was switched to cabozantinib. Six weeks later, he presented to the hospital with progressively worsening dyspnea. He was found to have obstructive shock secondary to cardiac tamponade. Emergent pericardiocentesis was performed, and 1 L of bloody fluid was drained with subsequent improvement in his blood pressure. Cytology was negative for malignant cells. The hospital course was complicated by NSTEMI (non-ST-segment elevation myocardial infarction) with troponin levels up to 6.44 (<0.03 ng/mL). He subsequently developed acute hypoxemic respiratory failure in the setting of decompensated congestive heart failure and possible pneumonia requiring intubation and mechanical ventilation. Given his overall prognosis, the decision to withdraw care was made and he passed away (Table 1).

**Table 1:** Timeline of the patient's clinical course. RCC, renal cell carcinoma; LVH, left ventricular hypertrophy

Time	Events
2012	Diagnosed with RCC and right radical nephrectomy was performed.
2014	Metastatic RCC, enrolled on a trial with atezolizumab and bevacizumab with initial partial response.
October 2017	Progression of disease, initiation of anti-LAG3 antibody plus nivolumab on a clinical trial.
January 2018	Progression of disease and initiation of ipilimumab and nivolumab on a clinical trial.
June 2018	Progression of disease and continued treatment beyond progression.
July 2018	Admission for dyspnea. Echocardiography demonstrated new LVH with new pericardial effusion. MRI showed suspicious lesions in the ventricles, biopsy was not diagnostic.
Early August 2018	Started on cabozantinib.
Late August 2018	Worsening shortness of breath, cardiogenic shock due to cardiac tamponade followed by a complicated hospital course.
September 2018	Withdrawal of care

## Discussion

Although primary cardiac tumors are uncommon, cardiac metastases are found in 9% of autopsy where a primary tumor is found and in 14% of metastatic cancer [3]. Almost any tumor type has the ability to spread to the heart, but the three most common malignant neoplasms are carcinoma of the lung, esophageal carcinoma, and lymphoma [1]. In the United States, there are approximately 65,000 new cases and almost 15,000 deaths from RCC each year [4]. Around 16% of patients with RCC have metastatic disease at presentation [5]. The most common metastatic sites are the lungs, bones, liver, adrenals, and central nervous system [6]. Cardiac metastases from RCCs are rare, with incidences ranging from 1.3% to 4.2% [7]. The most common mechanism of cardiac metastasis from RCC is the extension of a tumor column to the vena cava as a luminal mass, with the growth along the caval wall into the right heart chambers [8]. The other pattern that is a likely mechanism responsible for RCC metastasis to the left ventricle is through the lymphatic vessels of the thorax, involving the carinal lymph nodes that collect the drainage from the posterior wall of the heart, and, then, through reversed lymphatic flow caused by metastasis to the nodes, metastatic RCC can spread to the pericardium and the left myocardium [9].

Clinical presentations of cardiac metastasis are highly variable and depend on the location and the extent of cardiac involvement. While they are mostly asymptomatic, hypertension is the most common cardiac presentation of RCC and occurs in 20%-37.5% of patients [10]. Pericardial involvement may cause pericardial effusion, pericarditis, or even life-threatening cardiac tamponade, whereas dysrhythmias and decreased cardiac output are manifestations of myocardial disease. Endocardial involvement may lead to outflow tract obstruction and embolic phenomena. EKG changes in patients with cardiac metastases are usually nonspecific but may show possible ventricular/supraventricular arrhythmias or conduction defects. Pericarditis is rarely accompanied by the typical ST-segment elevations. Not infrequently, only nonspecific ST-segment changes are found. Pericardial effusion can cause low voltage and electrical alternans [11].

Echocardiography is the diagnostic modality of choice for the initial evaluation of a patient with suspected cardiac metastasis. It is widely available and can simultaneously assess for other cardiac conditions. This patient’s new biventricular hypertrophy was the initial sign of cardiac involvement secondary to underlying metastatic RCC. Other myocardial infiltrative diseases such as myocarditis can present with similar findings. In patients on immune checkpoint inhibitors, the diagnosis of myocarditis needs to be entertained, as these patients are managed with immune suppression rather than anti-neoplastic drugs. Our patient’s myocardial biopsy was negative for inflammatory cells. It was also negative for amyloidosis. When masses in the heart are evaluated on echocardiography, the apex and base of the heart are difficult to evaluate due to foreshortening, and poor acoustic windows may limit visualization of the entire wall [12]. CT is a good modality for diagnosis of cardiac metastasis, as its large field of view and excellent spatial resolution result in high sensitivity for pericardial effusions and masses. The limitations of CT in the evaluation of cardiac metastasis are limited myocardial tissue characterization and ionizing radiation exposure [2].

CMR is the best imaging modality for evaluating cardiac masses and differentiating inflammation from cardiac metastases. Moreover, it can distinguish between thrombus and cardiac tumors and is an invaluable non-invasive method for differentiating malignant and benign cardiac tumors [13]. The imaging characteristics that are useful for the differentiation are tumor size, invasion of the free wall or adjacent structure, and first-pass perfusion to determine tumor’s vascularity [13]. Although in a majority of cases, diagnosis of cardiac metastasis can be made through imaging, tissue diagnosis remains the most definitive method to differentiate neoplastic from non-neoplastic masses and to guide treatment with either definitive or palliative therapy [14]. However, it is limited by tissue sampling error, as in our case.

During the last decade, the survival of patients with advanced RCC has significantly improved from a median overall survival of approximately 12 months in the cytokines era to more than 26 months with first-line vascular endothelial growth factor inhibitors [15]. Frontline immune-therapies have further improved overall survival. In a recent report of a randomized trial involving ipilimumab and nivolumab in the frontline setting, at 42 months just over half the patients remained alive [16]. With improved survival of patients treated with immune checkpoint inhibitors, coupled with the risk of autoimmune myocarditis, the possibility of cardiac involvement by tumor must be examined in patients with cardiac symptoms. Pericardial effusion can be due to hemorrhage from vascular tumors, as in this patient, or autoimmune pericarditis, which would be treated with steroids. Immediate pericardiocentesis is indicated for cardiac tamponade. Re-accumulation of pericardial fluid after drainage is expected in these cases. In an attempt to preserve cardiac function by preventing re-accumulation of pericardial fluid, treatments such as sclerotherapy, balloon pericardiotomy, pericardial window, and catheter placement have been implemented [12].

## Conclusions

We reported one of the rare cases of RCC with cardiac and pericardial metastases in a patient on immunotherapy. This case highlights the importance of cardiac imaging for patients with RCC who develop cardiac symptoms. Specifically, advanced cardiac imaging modalities such as CMR are helpful in evaluating inflammation due to immunotherapy and metastases to the myocardium.
